# How Variability and Effort Determine Coordination at Large Forces

**DOI:** 10.1371/journal.pone.0149512

**Published:** 2016-03-02

**Authors:** Michalis Kolossiatis, Themistoklis Charalambous, Etienne Burdet

**Affiliations:** 1 Department of Mathematics and Statistics, Lancaster University, Lancaster, United Kingdom; 2 Department of Signals and Systems, Chalmers University of Technology, Gothenburg, Sweden; 3 Department of Bioengineering, Imperial College of Science, Technology and Medicine, London, United Kingdom; VU University Amsterdam, NETHERLANDS

## Abstract

Motor control is a challenging task for the central nervous system, since it involves redundant degrees of freedom, nonlinear dynamics of actuators and limbs, as well as noise. When an action is carried out, which factors does your nervous system consider to determine the appropriate set of muscle forces between redundant degrees-of-freedom? Important factors determining motor output likely encompass effort and the resulting motor noise. However, the tasks used in many previous motor control studies could not identify these two factors uniquely, as signal-dependent noise monotonically increases as a function of the effort. To address this, a recent paper introduced a force control paradigm involving one finger in each hand that can disambiguate these two factors. It showed that the central nervous system considers both force noise and amplitude, with a larger weight on the absolute force and lower weights on both noise and normalized force. While these results are valid for the relatively low force range considered in that paper, the magnitude of the force shared between the fingers for large forces is not known. This paper investigates this question experimentally, and develops an appropriate Markov chain Monte Carlo method in order to estimate the weightings given to these factors. Our results demonstrate that the force sharing strongly depends on the force level required, so that for higher force levels the normalized force is considered as much as the absolute force, whereas the role of noise minimization becomes negligible.

## Introduction

Motor control involves the coordination of multiple effectors (muscles, joints and limbs) for the implementation of a task. Even the most basic movements, such as grasping and reaching, can be performed in many ways because the human body uses more degrees-of-freedom (DoF) than needed [1], since several effectors get involved, exceeding the dimensionality of the task requirements. However, several tasks are shown to be consistently implemented via a narrow set of options. Based on this observation, a fundamental research question is how the the central nervous system (CNS) selects a particular set of movements among the vast set of available options.

Several theories attempted to answer this question. Some of them suggest that there is an inherent set of constraints in the CNS such that certain combinations of effectors are stable for certain type of tasks (see, for example, [2–5] and references therein), thus restricting the options considerably and making the problem tractable. Other theories suggest that coordination is achieved among multiple effectors by means of setting common parameters during the planning process [6]. On the other hand, many behavioral goals were uniquely specified by defining a control policy emanating from the optimal solution of the minimization of some cost function, suggesting that the CNS performs some sort of optimal control (see, for example, [7]).

Approaches based on optimal control theory could replicate patterns of reaching movements observed in the human arm [8] as well as account for the structure of the force variability in finger movement [9]. There are several factors that affect motor coordination and for which optimal control has been proposed, such as jerk, torque, torque change and energy; however, recent works have shown that the most representative factors are (i) variability of motor output, and (ii) effort involved [7]. As pointed out in [10], the predictions concerning the temporal shape of the optimal movement of these two costs are similar. This is expected since the requirement to reduce variability under signal-dependent noise leads to a term in the cost function that penalizes the sum of squared motor commands over the movement, the same term usually used for penalizing the effort involved [11]. As a result, for motor behaviors with temporal redundancy, it is hard to dissociate the costs for variability of motor output and effort involved.

An experimental paradigm was developed in [10] in which the influence of these two factors could be dissociated by studying how subjects combined the total force of two fingers, one from each hand. As the deviation due to motor noise grows linearly but with a different gradient for each finger, it was possible to observe how the CNS considers effort and variability by combining different fingers. The results demonstrated that the absolute force determined how the subjects combined the fingers at over 70%, while the influence of the normalized force (i.e., the force divided by the maximum strength of the effectors) and force variability counted less than 15% each.

The question addressed in this study is whether the cost function determining how the effort is shared between the effectors varies with the applied force level. In particular, the highest force levels required in [10] was limited to 16 Newtons (N), while subjects can generally exert forces that may exceed 30N. Would the nervous system modify the way it considers the effort at high force levels? Our hypothesis is that during the preservation of a *relatively large* force level, the distribution of forces, when normalized for the individual maxima, become proportionate to their relative strengths. The rationale behind this hypothesis is that at large force levels people would aim to distribute the difficulty of the task proportionally to avoid uncomfortable situations where maximum effort is used by one part (here one of the fingers) while the other part expends little effort. To examine this hypothesis we carried out a similar experiment to that in [10], where we first confirmed the results for the small range of force used in that study. Extending the range of applied forces up to 28N (a level that was feasible for all participants), our results show that the participants largely changed their cost function with the required force level. In particular the normalized effort becomes a significant factor of effort sharing between the effectors.

## Methods

### Participants

In this experiment, 14 adults (on average 23 years old with standard deviation 4 years) without known sensorimotor impairment from the postgraduate population of Imperial College London participated. All participants were right handed and 4 of them were females. The experiment was specifically approved by the Ethics Committee of Imperial College London, and each subject was informed of the details of the procedure of this study and signed a consent form prior to starting it.

### Apparatus

Participants sat in front of a computer monitor with forearms supported on a flat desktop. Two force transducers (more specifically, we used Phidgets sensors, with maximum weight capacity of 20*kg* and repeatability error maximally 0.1*N*) integrated into keyboard buttons were placed on the table in front of the participant. The participant always kept their wrists on the table throughout whilst pressing the buttons.

**Fig 1:**
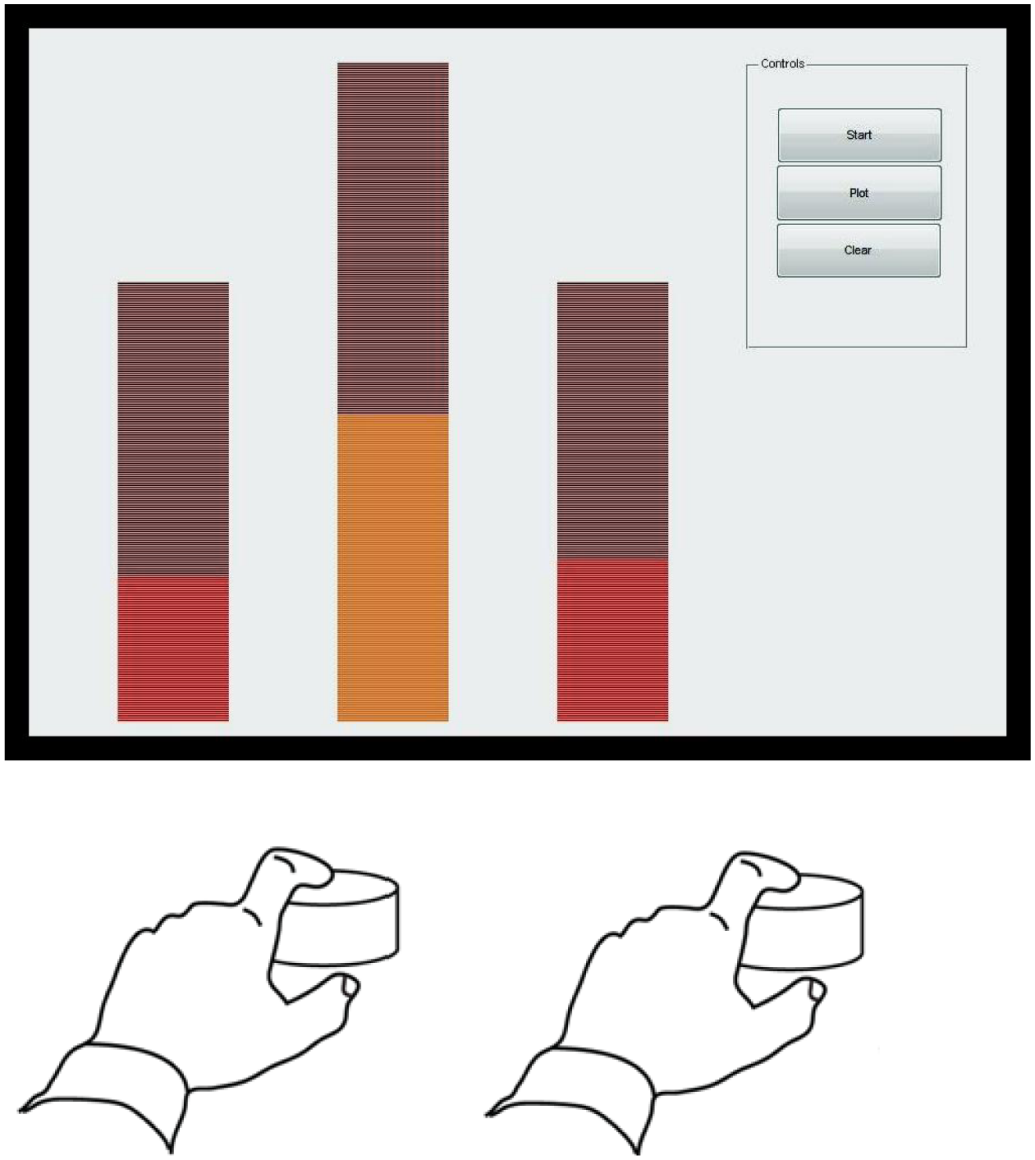
Experimental setup: participants pressed with a finger (index or little) of both left and right hands on isometric force transducers. The subjects were required to match the goal force level (a horizontal line in the central bar) as accurately as possible with the summation of each finger’s force level (summation of the force levels is given by the LEDs in the central bar and the individuals’ force levels of the left and right hand are given by the left and right bars, respectively).

Participants received continuous visual feedback about the total force produced as well as the individual absolute force levels via an array of LEDs, as shown in Fig 1. In contrast to [10], where only the sum of forces in the two fingers was displayed, in this experiment the individual forces were also displayed, as we wanted to investigate whether this would affect the cost function with respect to [10]. Note that the instructions were to concentrate on the total force produced. The target force level was represented on screen by two lines in either side of the total force bar, such that the participant could easily identify the target force and exactly determine his/her error throughout the process. Force was sampled at a rate of 200 Hz.

### Experimental trials

The trials were divided into 2 parts, the *unimanual* and the *bimanual* trials, each of which has been realized in consecutive days. In order to avoid fatigue, participants had regular breaks of about a minute between tasks; in addition, participants would take a break for a few minutes when changing fingers.

#### Unimanual trials

For the unimanual trials the force was recorded over 7*s* when trying to maintain forces from 2, 4, 6, … N and up until the force for which the participant can no longer maintain for 7*s*. The trials were executed for the index and little fingers of both right and left hands. The participants were to press and hold the force transducer and try to reach a line on the screen indicating they have reached the required force.

#### Bimanual trials

For the bimanual trials we recorded the forces over 7*s* for all 4 possible combinations of the index and little fingers, using a transducer for each finger. For all but the combination of both index fingers the participant tried to maintain forces of 2,4,6*N* and increased in 2N intervals until the participant’s maximum force was reached. For the combination of both index fingers the participant tried to maintain all forces for 7*s* up to force level of 28*N*. We did not exceed 28*N*, since not many participants could pass this limit and the data would thus be limited.

### Optimal control model

Let *x*_*i*_ denote the force of finger *i* with mean $E\left(x_{i}\right)\equiv u_{i}$ corresponding to the motor command; *k*_*i*_ is the coefficient of variation determined by minimizing the Mean Squared Error (MSE) for finger *i* over all forces (i.e., *σ*(*x*_*i*_) = *k*_*i*_ *u*_*i*_) and *MVC*_*i*_ is its maximum voluntary contraction. Finally, *g* denotes the target force level using both fingers. The association between *k*_*i*_ and *MVC*_*i*_ is weak (see subsection MVC measurement), so we include both these effects in the cost function. We use a method that attaches weights λ, *μ*, and *ν* to the cost functions that may affect the decision of the participants. These weights correspond to the non-normalized effort, the normalized effort and the squared error, respectively. Therefore, the cost function of each participant is the following:

$$\begin{array}{c} J=\tilde{\nu }E\left[{\left(x_{i}+x_{j}-g\right)}^{2}\right]+\tilde{\lambda }\left(u_{i}^{2}+u_{j}^{2}\right)+\tilde{\mu }({\left(\frac{u_{i}}{MVC_{i}}\right)}^{2}+{\left(\frac{u_{j}}{MVC_{j}}\right)}^{2}) \end{array}$$ (1)

$$\begin{array}{c} =\tilde{\nu }{\left(u_{i}+u_{j}-g\right)}^{2}+\tilde{\nu }k_{i}^{2}u_{i}^{2}+\tilde{\nu }k_{j}^{2}u_{j}^{2}+\tilde{\lambda }\left(u_{i}^{2}+u_{j}^{2}\right)+\tilde{\mu }({\left(\frac{u_{i}}{MVC_{i}}\right)}^{2}+{\left(\frac{u_{j}}{MVC_{j}}\right)}^{2}) \end{array}$$ (2)

$$\begin{array}{c} =\tilde{\nu }{\left(u_{i}+u_{j}-g\right)}^{2}+\underset{a_{i}}{\underset{︸}{\left(\tilde{\nu }k_{i}^{2}+\tilde{\lambda }+\frac{\tilde{\mu }}{{\left(MVC_{i}\right)}^{2}}\right)}}u_{i}^{2}+\underset{a_{j}}{\underset{︸}{\left(\tilde{\nu }k_{j}^{2}+\tilde{\lambda }+\frac{\tilde{\mu }}{{\left(MVC_{j}\right)}^{2}}\right)}}u_{j}^{2}, \end{array}$$ (3)

where

$$\begin{array}{ccccc} \tilde{\nu }=\frac{\nu }{b_{\nu }}=\frac{\nu }{E(k_{i}^{2}+k_{j}^{2})} & & \tilde{\lambda }=\frac{\lambda }{b_{\lambda }}=\frac{\lambda }{2} & & \tilde{\mu }=\frac{\mu }{b_{\mu }}=\frac{\mu }{E(1/MVC_{i}^{2}+1/MVC_{j}^{2})}, \end{array}$$

are the normalized weights (so that the estimates of λ, *μ*, and *ν* will be equal in the case the effect of the non-normalized effort, the normalized effort and the squared error on the optimal solution are the same).

In order to estimate the weight of the normalized expected error $\tilde{\nu }$, the coefficient of variation (*k*) for each finger of each participant is calculated (more details in subsection Noise measurement). In addition, in order to estimate the weight of the normalized effort cost $\tilde{\mu }$, the maximum voluntary contraction (MVC) for each finger of each participant is calculated (more details in subsection MVC measurement). In [10] based on the cost function used the optimal force command was found and it is given as a proposition below.

**Proposition 1**. *For finger i working with finger j, the optimal force command* $u_{i}^{*}$ *is given by*

$$\begin{array}{c} u_{i}^{*}=\frac{a_{j}}{a_{i}+a_{j}+a_{i}a_{j}/\nu }, \end{array}$$ (4)

*where ν is the weight of the expected squared error, and*

$$\begin{array}{c} a_{i}=\tilde{\nu }k_{i}^{2}+\tilde{\lambda }+\frac{\tilde{\mu }}{{\left(MVC_{i}\right)}^{2}}, \end{array}$$ (5)

*where* $\tilde{\nu },\tilde{\mu },\tilde{\lambda }$ *are as defined above*.

*Similar results hold for finger j when working with finger i*.

From the optimal force commands, the optimal force distribution is given in the corollary below.

**Corollary 1**. *For finger i working with finger j the optimal force distribution* $c_{ij}^{*}$ *is given by*

$$\begin{array}{c} c_{ij}^{*}=\frac{u_{i}^{*}}{u_{i}^{*}+u_{j}^{*}}=\frac{a_{j}}{a_{i}+a_{j}}. \end{array}$$ (6)

In the rest of this paper we focus on analyzing the force distribution for the combination of the left little and right index fingers (*i* and *j* in the above equations, respectively). The reason we focus on these is that this combination exhibits the largest difference in absolute individual performance of the two fingers, and it is therefore easier to distinguish between the effect of the normalised and the absolute force. However, analysis with other finger combinations showed similar tendencies.

Assume that we have *M* participants and for each force level *l*, we have *N*_*lm*_ observations from participant *m*, *m* = 1, 2, …, *M*. We denote these observations by *y*_*lm*_(*n*), *n* = 1, 2, …, *N*_*lm*_. Let **y**_*l*_ denote the data from all participants and $N_{l}=\sum_{m=1}^{M}N_{lm}$ denote the total data size, for force level *l*. Assuming Gaussian noise with variance $\sigma_{y,lm}^{2}$ for force level *l* and participant *m*, the log-likelihood of the force distribution **y**_*l*_, conditional on the parameters $\Theta =\{\lambda ,\mu ,\nu ,\sigma_{y,lm}^{2}\}$, is given by

$$\begin{array}{c} logL\left(y_{l}|\Theta \right)=-\frac{N_{l}}{2}\log\left(2\pi \right)-\frac{1}{2}\sum_{m=1}^{M}N_{lm}\log\left(\sigma_{y,lm}^{2}\right)-\frac{1}{2}\sum_{m=1}^{M}\sum_{n=1}^{N_{lm}}\frac{{\left(y_{lm}\left(n\right)-c_{ij,lm}^{*}\right)}^{2}}{\sigma_{y,lm}^{2}}\;, \end{array}$$ (7)

where $c_{ij,lm}^{*}$ is as in Corollary 1 for *i*, *j*: left little and right index finger, respectively, force level *l* and participant *m*.

Since cost functions are unit-less, and an overall scaling factor does not matter, we constrain the sum of the free parameters to be equal to 1, i.e.,

$$\begin{array}{c} \nu +\lambda +\mu =1. \end{array}$$ (8)

Therefore, we essentially have two free parameters (additional to the number of $\sigma_{y,lm}^{2}$ parameters). Without loss of generality, we assume that *ν*, λ are the free parameters and *μ* is derived from these two.

We assume a noninformative joint prior for *ν*, λ and *μ*, under condition Eq (8), as well as an improper prior for each variance $\sigma_{y,lm}^{2}$:

$$\begin{array}{c} \pi \left(\nu ,\lambda ,\mu \right)=\frac{1}{2}\cdot 1_{\left\{\nu +\lambda +\mu =1\right\}}\;, \end{array}$$ (9)

$$\begin{array}{c} \pi \left(\sigma_{y,lm}^{2}\right)\propto \sigma_{y,lm}^{-2}\;, \end{array}$$ (10)

where 1_{*A*}_ is the indicator function that takes the value 1 if condition *A* is satisfied and 0 otherwise. The prior for $\sigma_{y,lm}^{2}$ corresponds to an inverse gamma distribution, *InvGa*(0,0) (a random variable *X* is said to follow an inverse gamma distribution *InvGa*(*α*, *β*) if its density function is given by $f_{X}\left(x\right)=\frac{\beta^{\alpha }}{\Gamma (\alpha )}x^{-(\alpha +1)}\exp\left\{-\beta /x\right\},\;x>0$). Although this prior is improper, the posterior distribution of $\sigma_{y,lm}^{2}$ is proper. We notice also that the case of *InvGa*(*α* = −1, *β* = 0), which corresponds to $\pi (\sigma_{y,lm}^{2})\propto 1$, as used in [10], was also implemented, with essentially identical results. Under the priors in Eqs (9) and (10), the joint posterior distribution of all parameters in each force level *l* is given up to a constant of proportionality by

$$\begin{array}{c} \pi \left(\nu ,\lambda ,\mu ,\sigma_{y,lm}^{2}|y_{l}\right)\propto \prod_{m=1}^{M}\sigma_{y,lm}^{-2-N_{lm}/2}\exp\{-\frac{1}{2}\sum_{m=1}^{M}\sum_{n=1}^{N_{lm}}\frac{{\left(y_{lm}\left(n\right)-c_{ij,lm}^{*}\right)}^{2}}{\sigma_{y,lm}^{2}}\}\cdot 1_{\left\{\nu +\lambda +\mu =1\right\}}\;. \end{array}$$

The assumption of having different variances for each participant and at each force level is a generalisation of the structure of [10], where it is assumed that at each force level all participant have the same variance (say, $\sigma_{y,l}^{2}$). See subsection Model fitting and comparisons for more details on this.

### Estimation method

Due to condition Eq (8) and the corresponding prior distribution of *ν*, λ, *μ*, these parameters are also dependent a posteriori, a fact that makes simulations more challenging. In order to circumvent this problem, we use a reparametrization *w*, *z* ∈ (0,1), where

$$\begin{array}{c} \begin{array}{c} w=\nu \\ z=\frac{\lambda }{1-\nu }\\ \mu =1-\nu -\lambda \end{array}\}\;\;⟺\;\;\{\begin{array}{c} \nu =w\\ \lambda =(1-w)z\\ \mu =(1-w)(1-z) \end{array} \end{array}$$ (11)

It is easy to show that the corresponding priors for *w* and *z* are independent *U*(0,1). The posterior distribution of $w,z,\mu ,\sigma_{y,lm}^{2}$ is therefore

$$\begin{array}{c} \pi \left(w,z,\mu ,\sigma_{y,lm}^{2}|y_{l}\right)\propto \prod_{m=1}^{M}\sigma_{y,lm}^{-2-N_{lm}/2}\exp\{-\frac{1}{2}\sum_{m=1}^{M}\sum_{n=1}^{N_{lm}}\frac{{\left(y_{lm}\left(n\right)-c_{ij,lm}^{*}\right)}^{2}}{\sigma_{y,lm}^{2}}\}\cdot 1_{\left(0<w<1\right)}\cdot 1_{\left(0<z<1\right)}\cdot 1_{\left(\mu =(1-w)(1-z)\right)}, \end{array}$$ (12)

where $c_{ij,lm}^{*}$ is now expressed using *w*, *z* and *μ*. As in [10], the force distributions were measured quite reliably, with *SE* = 0.0176*N*.

Markov chain Monte Carlo (MCMC) methods provide an approach to simulating the posterior distributions in complex multi-parameter problems without resorting to a search for the maximum likelihood solution. The posterior distribution Eq (12) is not of standard form, so in order to take samples from it we use MCMC methods, and in particular *Metropolis-within-Gibbs* [12]. For this algorithm we need to calculate the full conditional distributions (i.e., the distributions conditional on the data and all the other parameters) of each parameter in this model (except from *μ*, which can be deterministically calculated from *w*, *z*). For force level *l*, we have:

$$\begin{array}{c} \pi \left(w|y_{l},z,\sigma_{y,lm}^{2}\right)\propto \exp\{-\frac{1}{2}\sum_{m=1}^{M}\sum_{n=1}^{N_{lm}}\frac{{\left(y_{lm}\left(n\right)-c_{ij,lm}^{*}\right)}^{2}}{\sigma_{y,lm}^{2}}\}, \end{array}$$

where *w* appears in the expression for $c_{ij,lm}^{*}$. Similarly,

$$\begin{array}{c} \pi \left(z|y_{l},w,\sigma_{y,lm}^{2}\right)\propto \exp\{-\frac{1}{2}\sum_{m=1}^{M}\sum_{n=1}^{N_{lm}}\frac{{\left(y_{lm}\left(n\right)-c_{ij,lm}^{*}\right)}^{2}}{\sigma_{y,lm}^{2}}\}, \end{array}$$

where *z* appears in the expression for $c_{ij,lm}^{*}$, and

$$\begin{array}{c} \pi \left(\sigma_{y,lm}^{2}|y_{l},w,z\right)\propto \sigma_{y,lm}^{-2-N_{lm}/2}\exp\{-\frac{1}{2}\sum_{n=1}^{N_{lm}}\frac{{\left(y_{lm}\left(n\right)-c_{ij,lm}^{*}\right)}^{2}}{\sigma_{y,lm}^{2}}\}. \end{array}$$

The full conditional distribution of $\sigma_{y,lm}^{2}$ is simply an inverse gamma distribution,

$$\begin{array}{c} \sigma_{y,lm}^{2}|y_{l},w,z∼\text{InvGa}(N_{lm}/2,\sum_{n=1}^{N_{lm}}{\left(y_{lm}\left(n\right)-c_{ij,lm}^{*}\right)}^{2}/2), \end{array}$$ (13)

so we can directly simulate from it.

Unlike $\sigma_{y,lm}^{2}$, the posterior distribution of *w* and *z* are not of standard form. We therefore use independent Random Walk Metropolis Hastings (RWMH) updating steps for these parameters. In order to assure good mixing for these steps, we use the Adaptive RWMH method of [13].

To sum up, the full MCMC procedure for each force level *l* (we note that this algorithm is performed for each force level independently) is as shown in Algorithm 1.

**Algorithm 1** MCMC procedure for each force level

**Initialization**: We give appropriate, arbitrary starting values for all the parameters (*y*, *z*, *μ* and $\sigma_{y,lm}^{2}$).

  1. For *T* cycles, we iteratively:

    (a) simulate *w*, given the values of all the other parameters and the data, using a RWMH step.

    (b) simulate *z*, given the values of all the other parameters and the data, using a RWMH step.

    (c) calculate *μ* = (1 − *w*)(1 − *z*).

    (d) simulate $\sigma_{y,lm}^{2}$ for each *m*, given the values of all the other parameters and the data, from Eq (13).

  2. We discard the initial tail of the chain as a *burn-in* period. The values of the parameters in the rest of the chain correspond to random samples from their corresponding posterior distributions.

**Output**: By acquiring a sample for *w*, *z* and *μ*, we can then use Eq (11) to obtain samples for *ν*, λ and *μ*.

## Results

### Noise measurement

As already mentioned in the “Optimal control model” subsection, the force of each finger *x*_*i*_ was modeled as a random variable with a mean equal to the motor command (i.e., $E\left(x_{i}\right)=u_{i}$) and a noise with a standard deviation proportional to the motor command (i.e., *σ*(*x*_*i*_) = *k*_*i*_ *u*_*i*_), with *k*_*i*_ being the coefficient of variation determined by minimizing the Mean Squared Error (MSE) for each finger over all force levels. The instructed task (enforced by the feedback on the screen) was to minimize the error between produced and required force.

In our experiments, the coefficient of variation was computed by robust regression estimates via iteratively re-weighted least squares with a bi-square weighting function [14–17]. The results for all fingers are depicted in Table 1. The values of the mean and standard deviation for *k* are slightly higher than those in [10] consistently for all fingers. This may stem from the slightly different setup used in the two experiments, or by individual differences.

**Table 1:** Mean and standard deviation over all participants for the coefficient of variation measured in our experiments and in [10]. All measurements are in Newtons (N).

	Left index	Right index	Left little	Right little
Mean *k* (%)	1.28	1.62	2.38	1.79
Standard Deviation *k* (%)	0.63	0.59	1.2	0.75
Mean *k* (%) [10]	1.11	0.82	1.61	1.31
Standard Deviation *k* (%) [10]	0.4	0.26	0.66	0.46

### MVC measurement

The mean and standard deviation of the maximum voluntary contraction (MVC) found for all fingers are depicted in Table 2.

**Table 2:** Mean and standard deviation of MVC measured in our experiments and in [10]. All measurements are in Newtons (N).

	Left index	Right index	Left little	Right little
Mean MVC	20.43	23.29	12.71	13.43
Standard Deviation MVC	4.09	5.18	2.30	2.87
Mean MVC [10]	34.33	36.94	17.74	19.93
Standard Deviation MVC [10]	10.50	8.72	7.58	5.59

Note that the MVC was measured in a different way in the two papers. In our experiment, the subjects had to reach their MVC at the end of that part of the session and they had to maintain this for 7*s*. In [10], the mean of the highest 5% of the samples was determined for each trial, and the highest score of the three available trials was taken as the MVC measurement for the finger. These differences may explain why the mean value of MVC was higher in the previous paper.

**Fig 2:**
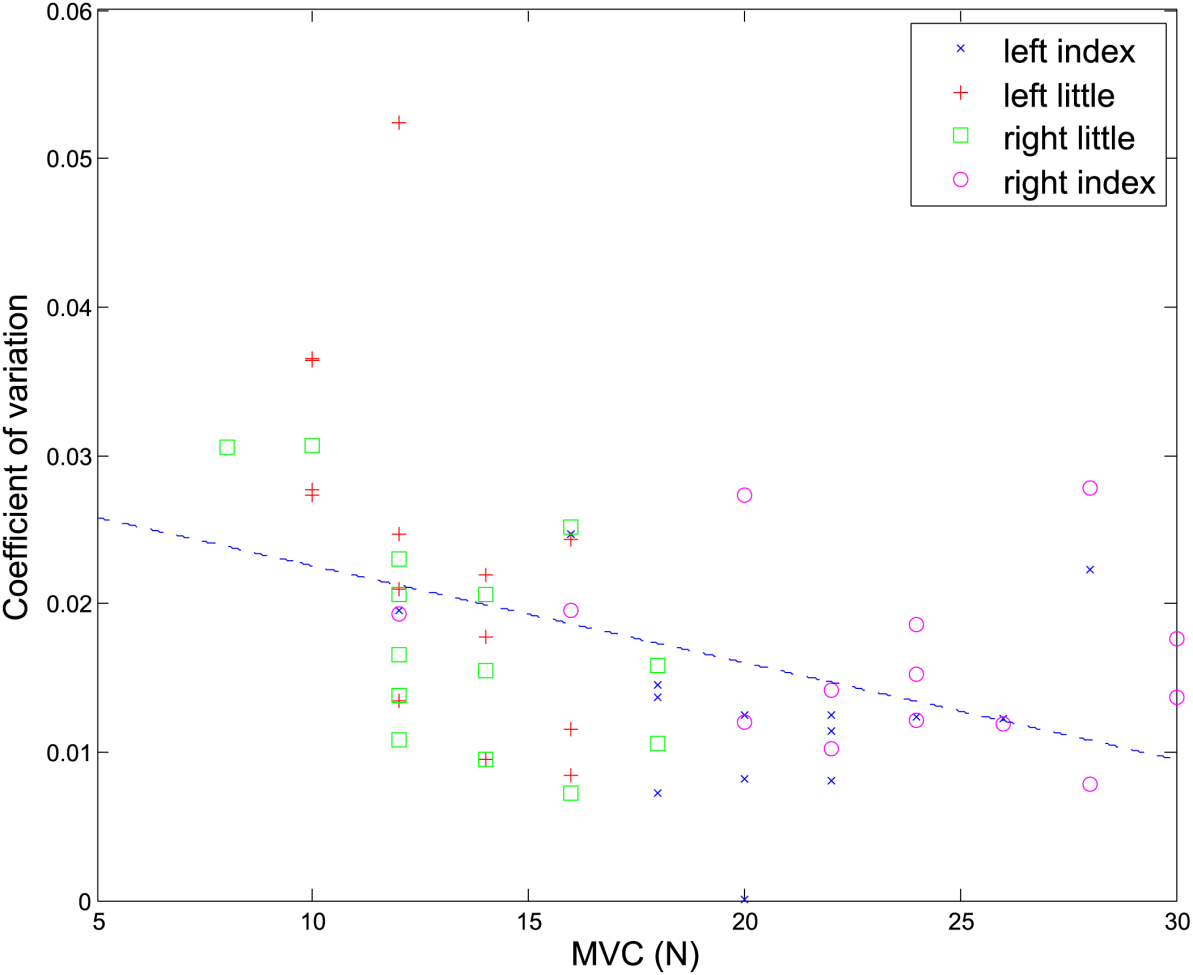
Coefficient of variation (*k*) versus maximum voluntary contraction (MVC) for all fingers and participants. The dashed line is the line of least-squares fit for the whole data set.

Fig 2 shows the correlation between *k* and MVC for all fingers and all participants. The dashed line indicates the fitted linear regression line, when all data are considered. It is clear from this figure that there is a negative relationship between the two quantities. However, this relationship, as indicated by the small slope of the fitted line (−0.00065), although significant (*t*(54) = −3.42, p-value = 0.0012), is not particularly strong. Moreover, the coefficient of correlation between *k* and *MVC* (*r* = −0.4224) also indicates a weak relationship between the two (the value of *r* found is also very close to the value found in [10]). As a result, it is sensible to include variability as an additional term in the cost function.

### Model fitting and comparisons

For each force level the above algorithm was implemented with *T* = 90000 iterations, from which the first 40000 samples were discarded as burn-in.

**Fig 3:**
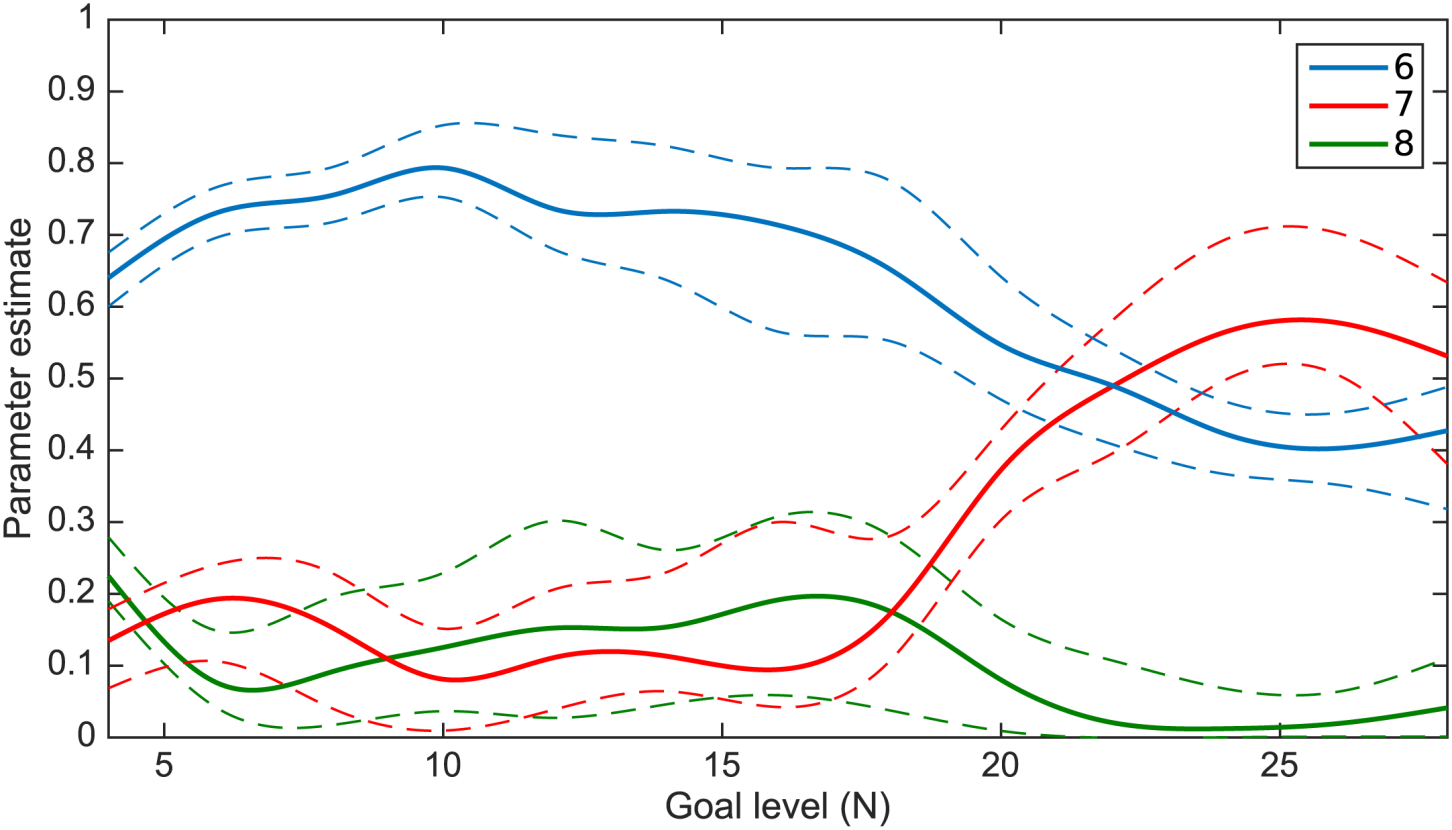
Posterior medians and 95% credible intervals for the parameters *ν*, *μ* and λ, for each force level. The continuous lines present the posterior median for each parameter, whereas the dashed lines show 95% credible intervals.

Fig 3 exhibits the results for *ν*, *μ* and λ. The continuous lines present the posterior median for each parameter, whereas the dashed lines show 95% credible intervals (we also computed posterior means, instead of medians, with identical results). The results in Fig 3 show that *for small force levels* the weight attributed to the absolute effort (λ) is much larger than the weight for the normalized effort (*μ*). This is consistent with the findings in [10], who studied this behavior up to force level equal to 16N. However, as the force level increases, the difference between these two weights diminished and at about 20N and higher, these weights become more or less equal. The results justify our hypothesis that as the task becomes more difficult, the weight for the normalized effort increases. Finally, the weight attributed to the squared error (*ν*) is generally small for all force levels.

**Fig 4:**
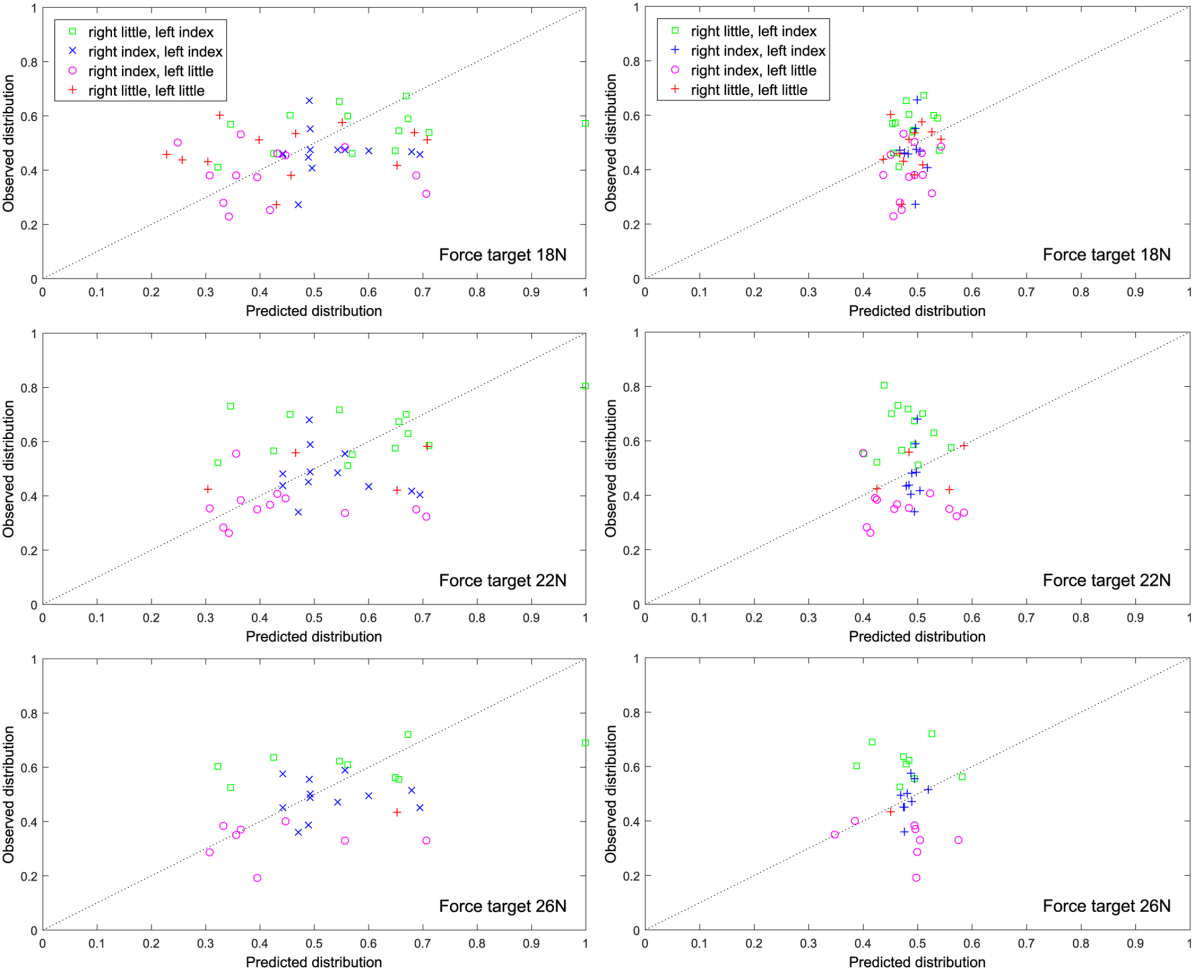
Fitted (x-axis) versus produced forces (y-axis) based on the assumption that only variability was optimized (left) and under the best fitting model (right) and force target 18N (top), 22N (middle) and 26N (bottom).

Fig 4 shows the optimal solution for fitted (x-axis) versus produced forces (y-axis) based on the assumption that only variability was optimized (left) and under the best fitting model (right) and force target 18N (top), 22N (middle) and 26N (bottom). It is evident that the variability-only cost function performed worse than the one that includes all effort and variability terms in predicting the chosen distribution, as the points in these graphs (actual pairs of fitted-produced forces) are not so close to the dotted line (which corresponds to the ideal scenario that the predicted and the observed forces coincide).

In order to verify that all three terms contribute significantly to the fit, we also fitted all models with only one term and all models with pairs of terms included. This is achieved by setting the value of some of *μ*, *ν*, λ equal to 0 and fitting the new model to the data.

As a first measure of model comparison, we calculated the log-likelihood of the data, averaged over all MCMC samples, under each model. This is a measure of model fit, with higher values indicating better fit. The results are shown in the first row of Table 3. The full model (i.e., the model with all *ν*, *μ* and λ included) provides the best fit and the model closest to that was the model with only *μ* and λ included (the model with *ν* = 0). This is consistent with the results above, since the weight *ν* is the smallest of the three for most force levels. In order to see if the difference between the two models is significant, we use Bayes factors (see, for example, [18]). The Bayes factor for two models, say *M*_1_, *M*_2_, is defined as

$$\begin{array}{c} B=\frac{L(y|M_{1})}{L(y|M_{2})}, \end{array}$$

where *L*(**y**|*M*) is the likelihood under model *M* and **y** is the full data set (including data from all participants and all force levels). For the two best fitting models stated above, $B=\frac{L(y|M_{\nu ,\mu ,\lambda })}{L(y|M_{\mu ,\lambda })}=e^{70.11}$, indicating very strong preference for the full model.

**Table 3:** Marginal log-likelihood and Akaike Information Criterion (AIC) for the full and all alternative models, for right index-left little combination.

Model	Full (*μ*, *ν*, λ)	*μ*, λ	*μ*, *ν*	*ν*, λ	*μ*	λ	*ν*
Log-likelihood	1265.16	1195.05	590.87	1076.37	367.04	751.36	295.92
AIC	-2547.61	-2421.34	-1208.12	-2254.51	-715.22	-1426.61	-554.18

Additionally, we also consider the Akaike Information Criterion (AIC) (see, among others, [19]):

$$\begin{array}{c} \text{AIC}=-2\hat{logL}\left(y\right)+2d, \end{array}$$ (14)

where $\hat{logL}$ is the maximum log-likelihood and *d* is the number of free parameters in the model. This statistic therefore takes into account both the model fit (measured by $\hat{logL}\left(y\right)$) and the model complexity (measured by *d*, and penalizing models with higher number of parameters). A lower value of AIC indicates a better fitting model. The results for AIC (Table 3, second row) indicate that the full model, despite having more parameters than the simpler models, is still the preferred one, by a large margin.

In conclusion, both model comparison measures used indicate that the model including all terms is the model that best describes the behavior of the data. All three terms have been demonstrated to be significant in explaining how the CNS assigns the task among the effectors when trying to reach a specific force target.

For comparison purposes we repeated the above analysis for the case in which all participants have the same error variance within each force level, as in [10] (in other words, having the same $\sigma_{y,l}^{2}$ for each participant instead of having $\sigma_{y,lm}^{2}$ for each participant *m*). We found that the behavior of the weights *μ*, *ν* and λ was in accordance to the one found before: at lower force targets the parameter λ is quite large, whereas the other two are small. Then, as the target increases, the value of *μ* increases and that of λ decreases, whereas *ν* stays at low values. In other words, the change in the effect of the two effort and the variability terms as we increase the force target, seems to be robust to the specification of the variances. On the other hand, when comparing the two models (using both the log-likelihood and the AIC), the model with different variances had a clear advantage over the model with the same variance for each force level, so using the model shown here is justified.

## Discussion

Our experiment extends the research on how efforts (normalized or absolute) and variability costs affect the way our CNS distributes work across different effectors in order to implement a task which has numerous alternative ways to be implemented. The influence of a finger’s individual maximum maintainable force was investigated with respect to the contribution of each finger in a dual finger task (one force target shared between two fingers). The main question addressed is the following: is the distribution of forces, when normalized for the individual maximums, proportionate to their relative strengths?

In summary, we show that during the preservation of a *relatively large* force level by the combination of a strong finger and a weak finger, for example an index finger and a little finger, the little finger’s force gradually reduces and the stronger finger’s force is increased in an anticipatory manner. As a result, it is concluded that over a range of target forces the distribution of effort changes. For a combination of little and index fingers, the little finger provides most absolute force and normalized force to start with, the proportion of its maximum force increases more than that of the index finger, up until the dual target becomes the maximum individual force for the index finger. 50% of each of the fingers’ maximum forces are being used above this point, suggesting that the sharing of effort is being utilized to minimize effort and strain on each finger, as well as variability of the force output over time.

The participants were asked to consider the middle column depicting the total force level, while we allowed them to have visual feedback of the force levels of the individual fingers as well. This feature was not present in the experimental setup of [10] and one could suspect that the participants would aim to equalize the two side columns, i.e., to aim at maintaining the normalized effort only. However, this did not occur; the results of [10] have been justified at low force levels and at high force levels it appears that it did not prohibit the increase of the normalized effort. It is possible that without the two extra columns the increase in the normalized effort could be more immediate or even dominate. Hence, the extra columns probably made the transition smoother, but did not prevent the normalized effort from becoming of significant importance.

## Supporting Information

S1 Dataset The data used in this experiment appear in zipped form in S1 Dataset. (ZIP)
